# Supplementary material for: Flexible iontronics based on 2D nanofluidic material
Source: Nat Commun. 2022 Aug 24;13:4965. doi: 10.1038/s41467-022-32699-x (PMC9402920; doi:10.1038/s41467-022-32699-x)
Supplement: Supplementary file 1 — Supplementary Information [file 41467_2022_32699_MOESM1_ESM.pdf]

Supplementary information for

Flexible iontronics based on 2D nanofluidic material

Di Wei[1*], Feiyao Yang[1], Zhuoheng Jiang[1], Zhonglin Wang[1,2*]

[1]Beijing Institute of Nanoenergy and Nanosystems, Chinese Academy of Sciences, Beijing 101400, P. R. China

[2]School of Materials Science and Engineering, Georgia Institute of Technology, Atlanta, GA 30332, USA

*Corresponding authors: Di Wei (weidi@binn.cas.cn)

Zhonglin Wang (zlwang@binn.cas.cn)

This file includes:

Supplementary Discussion

Supplementary Tables 1-3

Supplementary Figs. 1-14

Supplementary Discussion

Determination of Voc for the planar osmotic power source (Au/$AgNO_3$/GO/rGO/Au)

The planar osmotic power source is an open system, and water or oxygen from the environment might participate in the redox reactions. Theoretically, the Nernst equation is used to calculate the voltage introduced by redox reactions. However, since our device is operating as a solid-state power source, the potential could only be estimated with the guide of the Nernst equation as below.

$Ag^+ + e^- \rightleftharpoons Ag$ (1) $E^0_c = 0.80$ V

$4OH^- - 4e^- \rightleftharpoons O_2 + H_2O$ (2) $E^0_a = 0.40$ V, pH=14

$E_{redox} = E^0_c - E^0_a = 0.4$ V

E_{redox} of 0.4 V was estimated from the redox reactions above. Due to the large amount of KOH intake and unimpeded water permeation, highly concentrated solutions that were close to saturation were formed within rGO, maintaining a large ion concentration gradient and enabling ultrafast ion permeation of hydrated K^+ from rGO to GO driven by the ion gradient. The planar osmotic power source is in a solid-state form and it is impractical to calculation the concentration, however, the open circuit voltage of 1.2 V (Supplementary Fig. 2a) from Au/GO/rGO/Au power source came mainly from E_{diff} by the high K^+ concentration gradient since there were no redox reactions in it. The V_{OC} of the Au/$AgNO_3$/GO/rGO/Au power source is composed of the voltage from ion gradient diffusion (E_{diff}) and the redox reactions (E_{redox}) at the Au charge collector interface.

$V_{OC} = E_{diff} + E_{redox}$

Thus the theoretical V_{OC} could be calculated to be 1.6 V (V_{OC}= 1.2+ 0.4).

Determination of diffusion potential E_{diff} from ion gradient

As the negatively charged GO is cation-selective, it can transport cations (K^+) preferentially from the

high concentration rGO side to the low concentration GO side, generating the diffusion potential (E_{diff}). E_{diff} originates from the ion selectivity of the GO, which can result in differences in the diffusive fluxes of anions and cations. E_{diff} can be expressed as

$$E_{diff} = (t_+ - t_-)\frac{RT}{F}\ln\left[\frac{a_{high}}{a_{low}}\right]$$

where t_+ and t_- are the transference numbers for cation and anion, respectively. R, T, and F are the universal gas constant, absolute temperature, and Faraday constant, respectively. a_{high} and a_{low} are the activities of K^+ in the high concentration and low concentration sides, respectively. In the above equation, (t_+ - t_-) is called ion selectivity. It was reported that graphene nanopores[1] were found to preferentially transporting K^+ over its counter anions such as Cl^- with selectivity ratios over 100 and hydrated K^+ diffuses orders magnitude more quickly than most hydrated ions within the 2D nanofluidic channels. Based on such prior art[1], we calculated the ion selectivity of GO to K^+ is 0.99. The charge selectivity of GO to K^+ is very high (close to 0.99), and the E_{diff} for K^+ in the osmotic power sources (both the planar one and the 3D GO aerogel one) follow the same equation. The osmotic power source is operated as a solid state power source in contrast to those in traditional electrolyte. The measured E_{diff} is quite high, also indicating a very high selectivity of K^+ cations.

Calculation of the energy density of the planar osmotic power source

The energy density of the osmotic cell can be calculated by

$$E = I\int_0^t U$$

where I, U, t are the discharge current, the electric potential and discharge time, respectively.

The volumetric energy density (E_v) can be expressed as

$$E_v = E/Sh$$

where S, h represent the surface area and thickness of the GO device. From Supplementary Fig.1d, total

height of the power source is around 10 μm and surface area of the device is 0.6 cm × 0.2 cm. The energy from the first discharge was calculated by integration of area of the discharge curves (0.69 μWh) in Supplementary Fig. 3c. The maximum volumetric specific energy density of 6 mWh cm^{-3} was calculated. Maximum power of 3.39 μW can be calculated in Fig. 1e, and similarly the maximum volumetric specific power density of 28 mW cm^{-3} was obtained.

Calculation of areal power density of the 3D osmotic power source

The energy density of the osmotic cell can be calculated by

$$\mathrm{E} = \mathrm{I}\int_0^t U$$

where I, U, t are the discharge current, the electric potential and discharge time, respectively.

The areal energy density (E_s) can be expressed as

$E_s = E/S$

where S represent the surface area of the GO device.

The 3D osmotic source was made from the RTIL ionogel, which enables it made in a more compact space with area of 0.32 cm × 0.20 cm. The maximum power of 84 μW can be calculated from Fig. 4b and the areal power density is about 1.3 mW cm^{-2}.

Supplementary Table 1 Comparison of the EIS fitted results for the osmotic power source without RTIL (Au/$AgNO_3$/GO/rGO/Au) and with RTIL (Au/$AgNO_3$/GO/RTIL/rGO/Au).

	Solution resistance (Rs, Ω)	Charge transfer resistance (R_{ct}, Ω)
without RTIL	1.883×10^5	5.037×10^7
with RTIL	2.696×10^4	2.091×10^4

Supplementary Table 2 Summary of the properties of GO aerogels with different GO:LAA ratio.

GO:LAA	Raman I_D/I_G	Resistivity (Ω•cm)	Surface Area (m^2 g^{-1})	Pore size (nm)	V_{oc} (V)	I_{sc} (μA)
1:0	2.15	$>10^5$	403.6	1.22	0.333	0.8
1:0.1	1.93	$>10^5$	393.1	1.22	0.644	2.9
1:0.5	1.83	93.5	274.9	2.14	0.679	402
1:1	1.60	9.4	114.1	2.43	0.470	152
1:2	1.51	3.7	15.6	2.62	0.347	51.5

Supplementary Table 3 Comparison of the modular design osmotic power source output with various materials.

Type	Materials	Power density (mW cm^{-2})	Reference
This work	GO	1.3	
2D materials	GO	0.526	Ref. 2
	GO	0.077	Ref. 3
	GO	0.035	Ref. 4
	rGO	0.115	Ref. 5
	Mxene	0.21	Ref. 6
	Mxene/Kevlar	0.41	Ref. 7
Polymer	PES-Py/PAEK-HS	0.266	Ref. 8
	PMA/PS-b-P4VP	0.38	Ref. 9
	Polyimide	0.026	Ref. 10
Others	Silicon	0.77	Ref. 11
	SNF/AAO	0.286	Ref. 12
	Hydrogel/ANF	0.506	Ref. 13
	Mesoporous carbon/AAO	0.346	Ref. 14

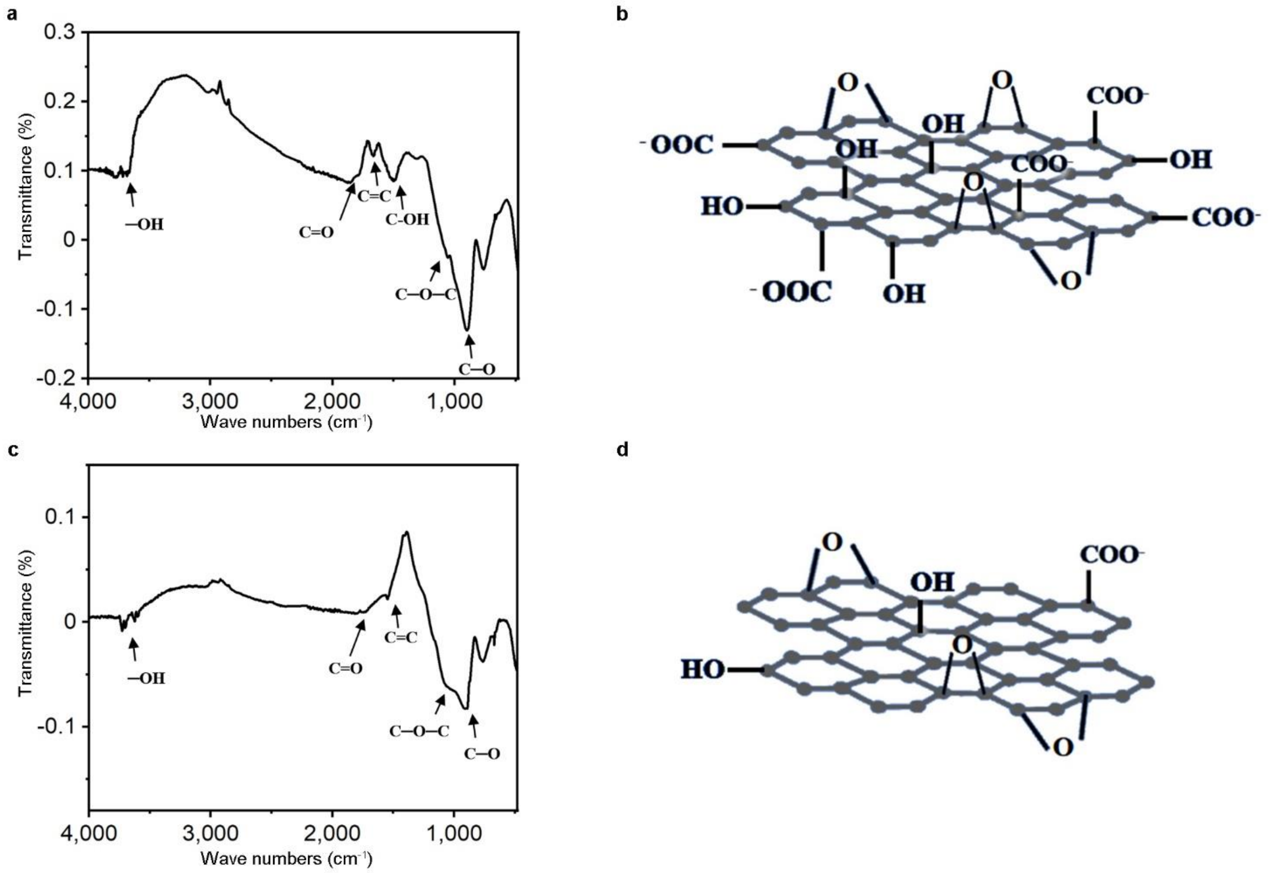

Supplementary Fig. 1 FTIR spectrum of GO and rGO with their empirical structural formula. **a**, FTIR spectrum of the GO. **b**, The empirical structural formula of GO. **c**, FTIR spectrum of the rGO. **d**, The empirical structural formula of rGO. FTIR spectrum of GO shows the characteristic peak at 3402 cm^{-1}, which corresponds to the hydroxyl group (-OH). The peaks at 1726, 1630, 1386, 1145 and 968 cm^{-1} correspond to the stretching vibration of C=O in the carboxyl group, C=C on the sp^2 carbon skeleton, C-OH group on the carbonyl group (-COOH), C-O-C group and C-O group on the epoxy group, respectively. FTIR spectrum also shows a reduction in the amount of hydroxyl and carboxyl groups in rGO. Source data are provided as a Source Data file.

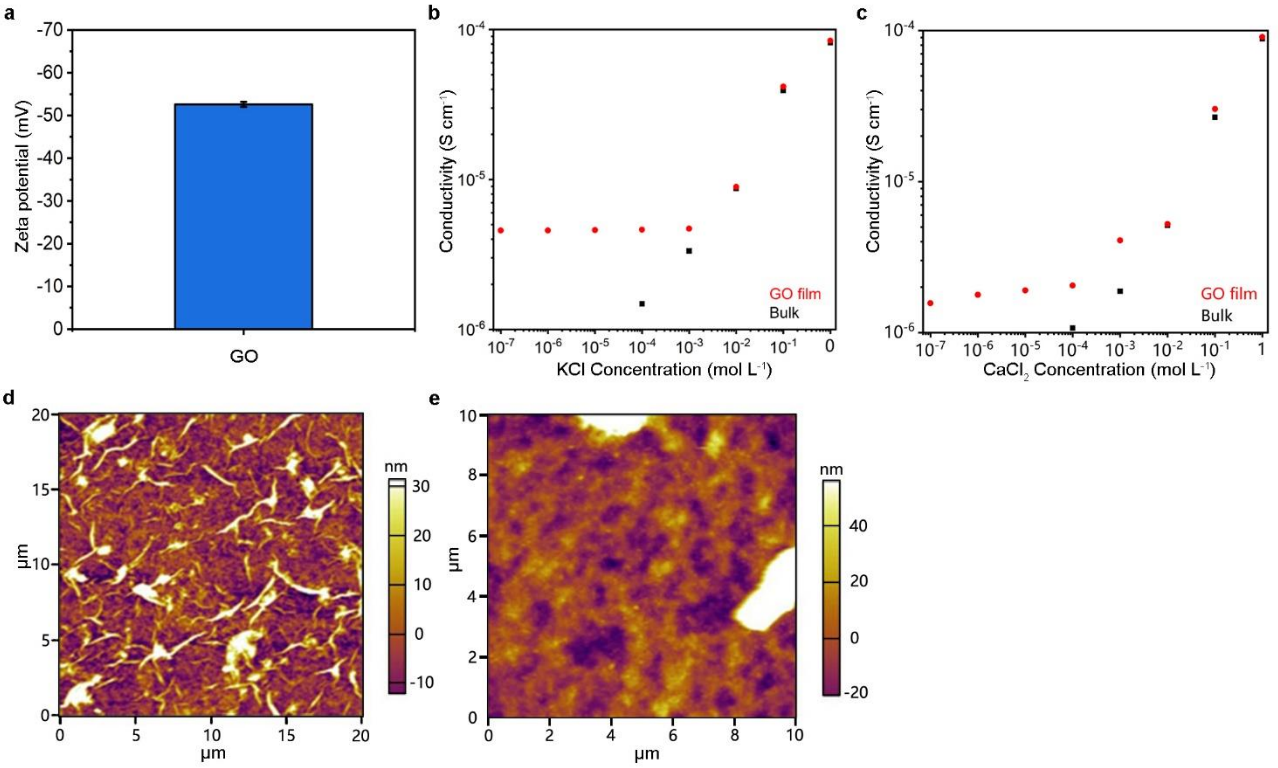

Supplementary Fig. 2 Characterization of the GO and rGO. a, Zeta potential of GO aqueous solution (5mg/mL, pH 4). Error bar represents standard deviations for 3 measurements. **b,** Ionic conductivity as a function of KCl concentration measured through 2D nanofluidic channels of GO. The conductivity of bulk solution is shown as benchmark. **c**, Ionic conductivity as the function of $CaCl_2$ concentration measured through 2D nanofluidic channels of GO. The conductivity of bulk solution is shown as benchmark. **d-e,** AFM on surface morphology of the GO film (**d**) and rGO film (**e**). Source data are provided as a Source Data file.

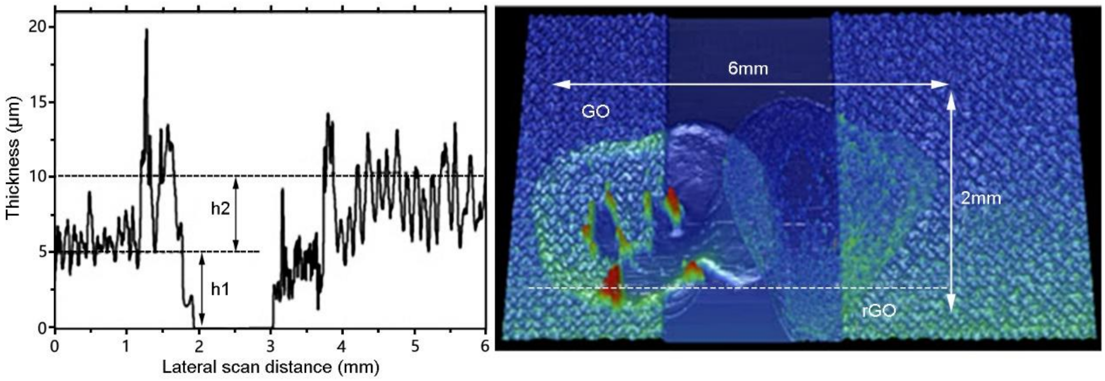

Supplementary Fig. 3 Surface profile of the osmotic power source. The height of the charge collector (h_1) and the GO/rGO coating (h_2) is respectively about 5 μm and 5 μm.

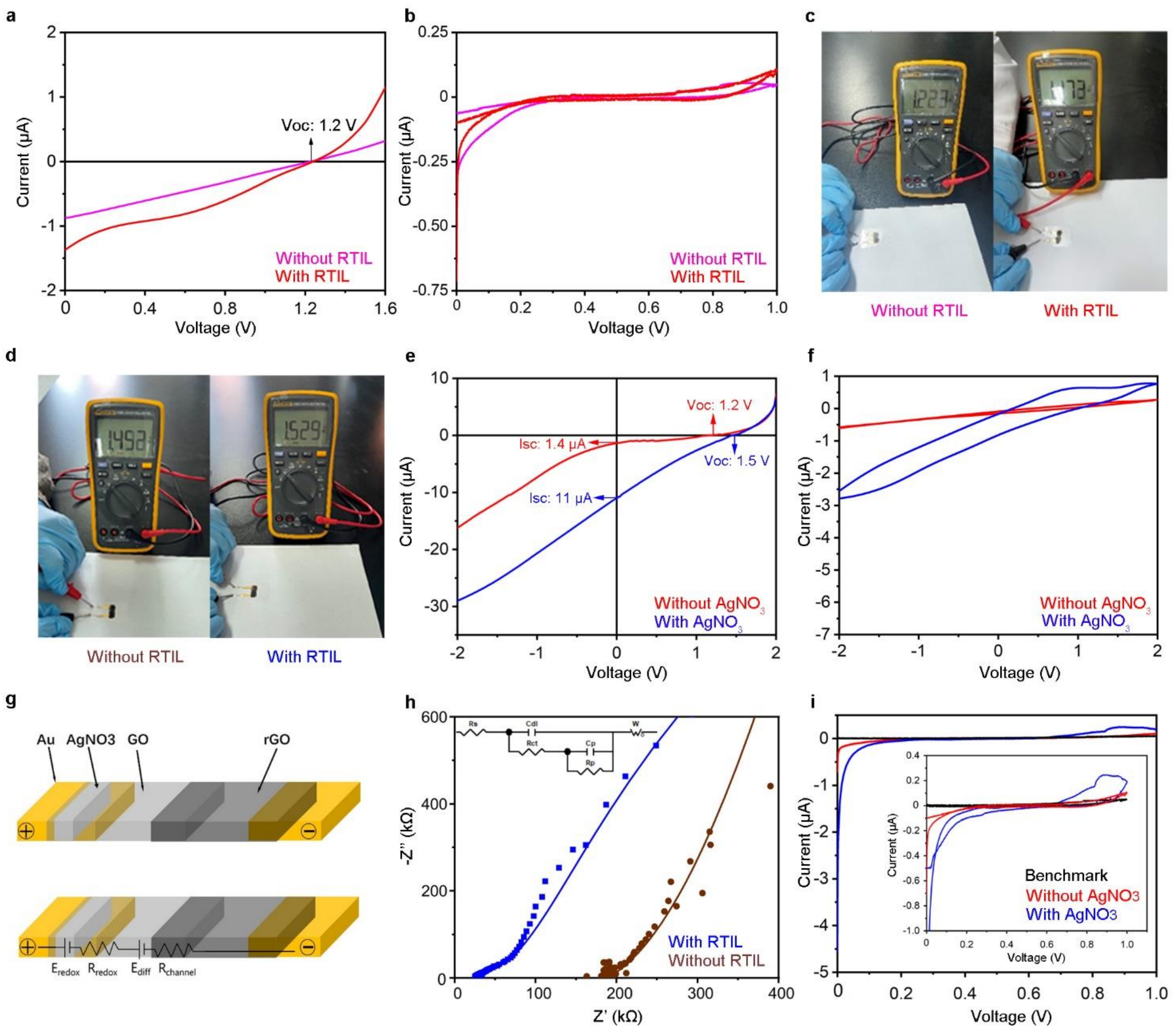

Supplementary Fig. 4 Electrochemical characteristics of the planar osmotic power source. a, I-V characteristics of the osmotic power sources without RTIL (Au/GO/rGO/Au) and with RTIL (Au/GO/RTIL/rGO/Au) at 25°C. **b,** Cyclic voltammogramme (CV) of the osmotic power sources without RTIL (Au/GO/rGO/Au) and with RTIL (Au/GO/ RTIL/rGO/Au) at the scan rate of 0.1 mV/s at 25°C. **c,** Voltage of the osmotic power sources without RTIL (Au/GO/rGO/Au) and with RTIL (Au/GO/RTIL/rGO/Au). **d,** Voltage of the osmotic power sources without RTIL (Au/$AgNO_3$/GO/rGO/Au) and with RTIL (Au/$AgNO_3$/GO/RTIL/rGO/Au). **e,** I-V characteristics of the osmotic power sources with $AgNO_3$ (Au/$AgNO_3$/GO/RTIL/rGO/Au) and without $AgNO_3$ (Au/GO/RTIL/rGO/Au). **f,** Cyclic voltammogrames of the osmotic power sources with $AgNO_3$ (Au/$AgNO_3$/GO/RTIL/rGO/Au) and without $AgNO_3$ (Au/GO/RTIL/rGO/Au) at 25°C at the scan rate of 10 mV s^{-1}. **g,** The structure and equivalent circuit of the osmotic power source (Au/$AgNO_3$/GO/rGO/Au). **h,** Nyquist plot of

electrochemical impedance spectrum (EIS) of the osmotic power sources with RTIL ($Au/AgNO_3/GO/RTIL/rGO/Au$) and without RTIL ($Au/AgNO_3/GO/rGO/Au$) at 25 °C under RH of 70%. The R_s (C_{dl}(R_{ct}(C_pR_p)) W equivalent circuit (Inset schematic) was used to fit the power source, where R_s is the solution resistance (contact resistance), C_{dl} is the double-layer capacitance, R_{ct} is the charge transfer resistance, C_p is the polarization capacitance, R_p is the polarization resistance and W is the Warburg impedance. **i,** The first scan in CV at the scan rate of 0.1 mV/s of different osmotic power sources: GO connecting both Au charge collectors as benchmark (Au/GO/RTIL/GO/Au), with $AgNO_3$ ($Au/AgNO_3/GO/RTIL/rGO/Au$) and without $AgNO_3$ (Au/GO/RTIL/rGO/Au). Inset picture shows the full cycle of complete CV. Source data are provided as a Source Data file.

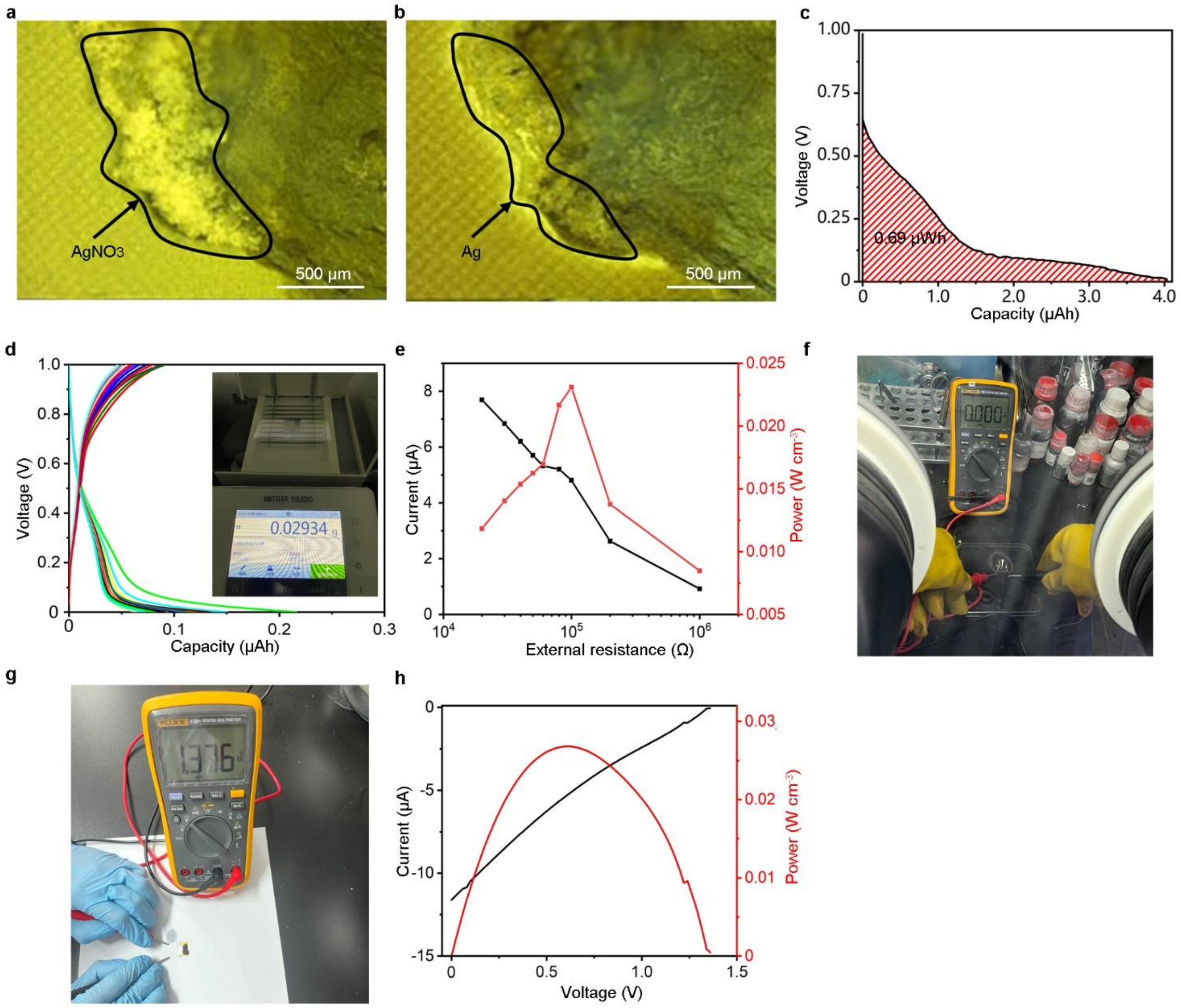

Supplementary Fig. 5 Characterization of planar osmotic power source ($Au/AgNO_3/GO/RTIL/rGO/Au$). a-b, The optical image of the $AgNO_3$/GO boundary before（**a**）and after（**b**）the power source was discharged. **c,** The energy capacity of the osmotic power source is 0.69 μWh calculated from the first discharge curve at 0.1 μA. **d,** The galvanostatic charge-discharge cycle performance of the osmotic power source. Inset picture shows its total weight of 30 mg. **e,** The output power density and current as the functions of load resistances and the output power density reaches a peak value of 23 mW cm^{-3} at the load resistance of ~100 kΩ. **f**, Voc of the device was zero in the glovebox (H_2O < 0.5 ppm). **g**, Voc of the power source that had been kept in glovebox for half year was measured at ambient environment. **h**, The I-V characteristics and power density of the power source that was kept in glovebox for half year. Source data are provided as a Source Data file.

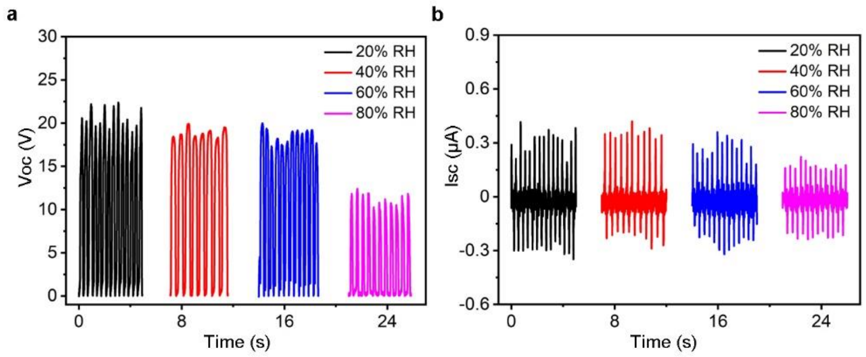

Supplementary Fig. 6 Output performance of the TENG pasted on skin under different relative humidity conditions. **a**, open-circuit voltage. **b**, short-circuit current. The TENG was driven by flapping the human skin to generate output electrical signals in the environmental simulation chamber (Vötsch Technik), and the impact flapping force exerted by the human hand on the TENG is not as identical as that exerted by the linear motor, resulting in the little difference in the output performance reported in Fig. 2c and 2d. Source data are provided as a Source Data file.

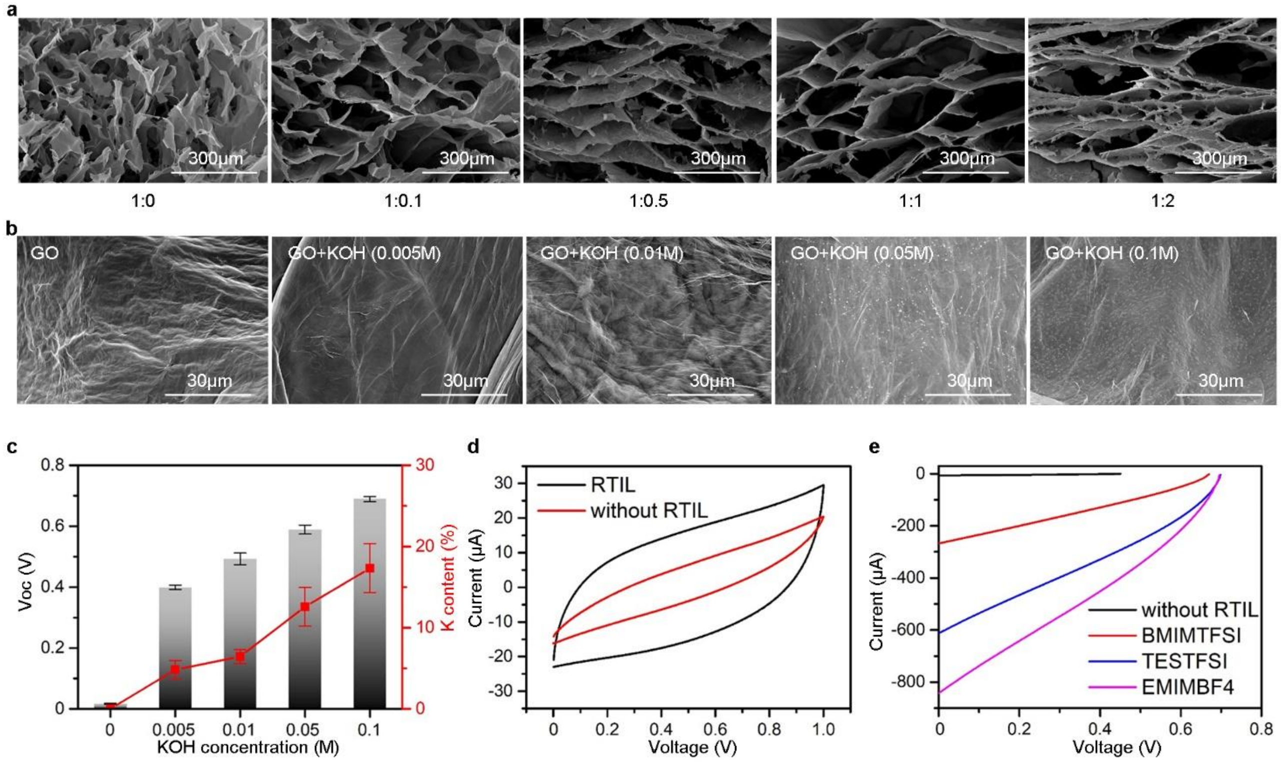

Supplementary Fig. 7 Characteristics of GO and GO+KOH aerogel. a, SEM images of GO aerogels with different GO:LAA ratio. **b**, SEM images of the GO+KOH aerogel with different KOH concentration. **c**, The relationship of the Voc of the power sources and the K^+ content in GO+KOH aerogels with increasing KOH concentration. **d**, CV of the power source with RTIL and the moisture-enabled power source without RTIL at 25 °C and RH of 70% at the scan rate of 0.1 mV s^{-1}. **e**, I-V characteristics of the power sources with different RTILs. Source data are provided as a Source Data file.

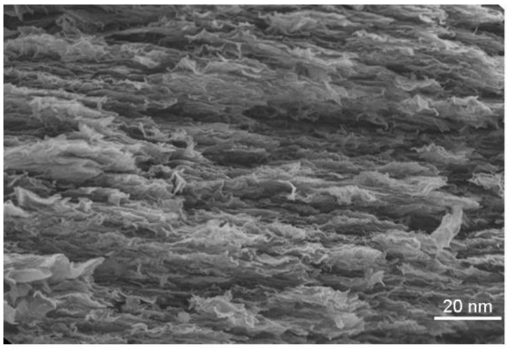

Supplementary Fig. 8 SEM image of the pressed GO aerogel (cross section).

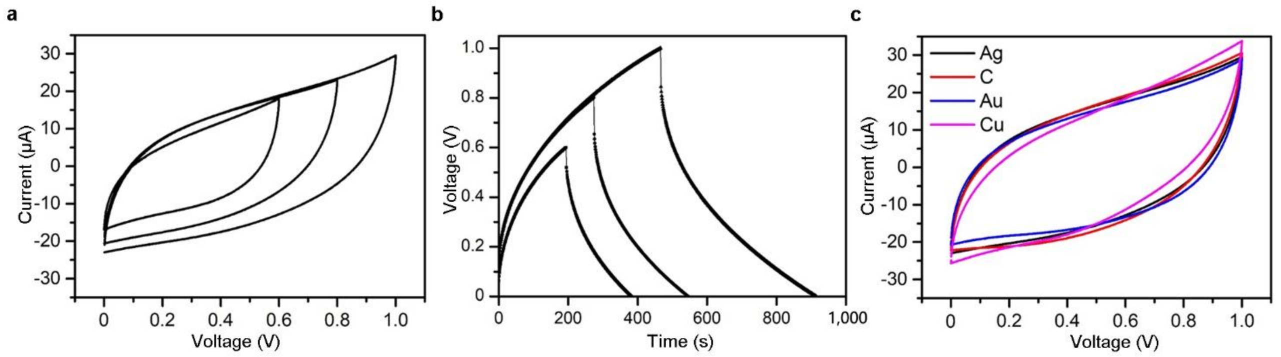

Supplementary Fig. 9 Electrochemical characteristics of GO aerogel osmotic power source. a, CV of the power source at the scan rate of 0.1 mV s^{-1}. **b**, The galvanostatic charge discharge tests of the power source at 0.1 mA. **c**, CV of the power source with different charge collectors (silver, carbon, gold, copper) at the scan rate of 0.1 mV s^{-1} at 25 °C. Source data are provided as a Source Data file.

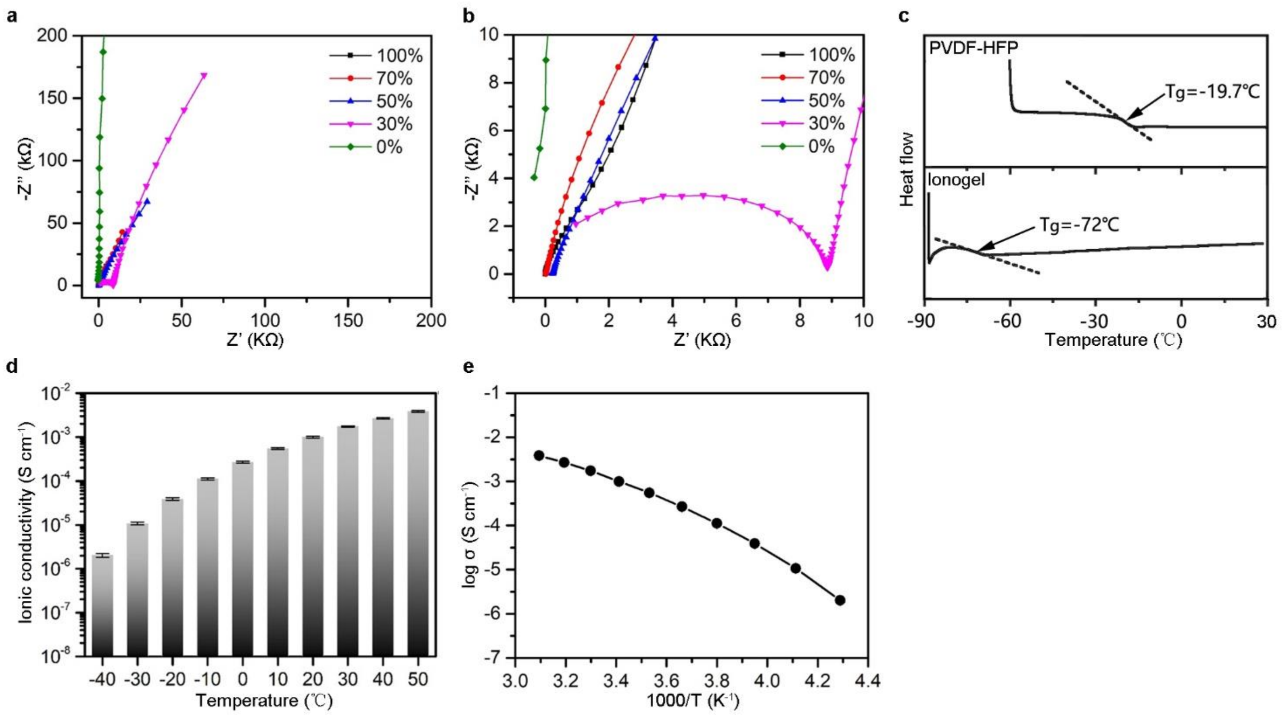

Supplementary Fig. 10 Characterization of the self-healing ionogel. a-b, The complex plane plot of the ionogels with different RTIL content at 25 °C. **c**, The glass transition temperature of the pure PVDF-HFP and the ionogel with 70 wt.% RTIL measured by DSC test. **d**, Temperature dependence of the ionic conductivity of the ionogel with 70 wt.% RTIL. **e**, Temperature dependence of the ionic conductivity of the ionogel with 70 wt.% RTIL. Source data are provided as a Source Data file.

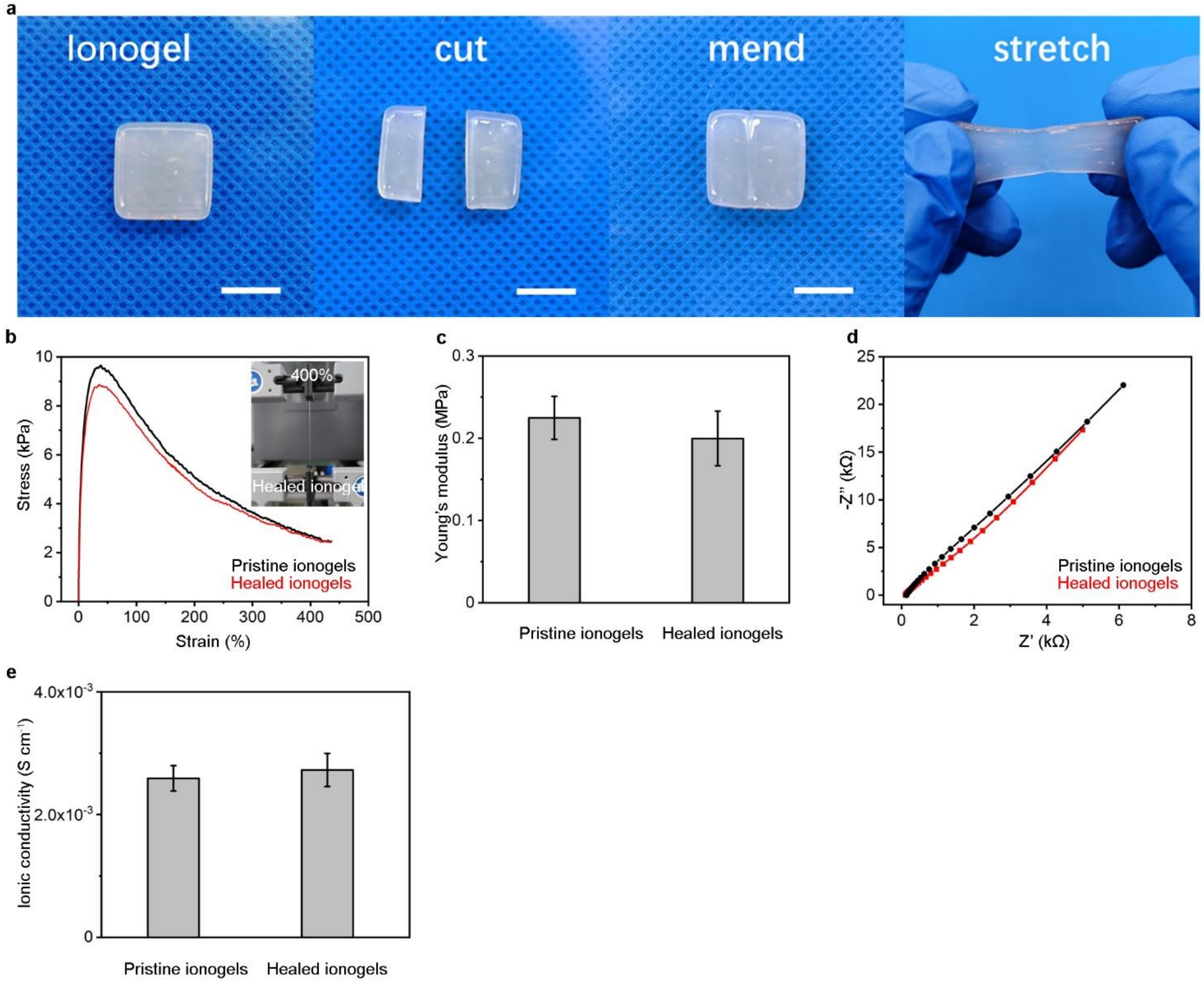

Supplementary Fig. 11 Mechanical and electrical properties of the self-healing ionogel. a, Photo of the self-healing demonstration of an ionogel at room temperature. **b**, Stress-strain curves of the original and healed ionogels. **c**, Young's modulus of the original and healed ionogels. Error bar represents standard deviations for 3 measurements. **d**, The complex plane plot of the ionogels before and after being cut. **e**, The ionic conductivity of the ionogels before and after being cut. Error bar represents standard deviations for 3 measurements. Source data are provided as a Source Data file.

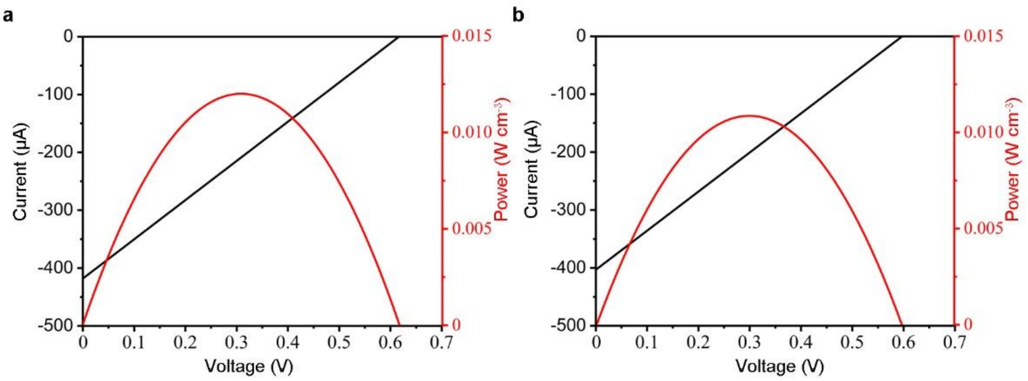

Supplementary Fig. 12 I-V characteristics and power density of the modular design power source. **a**, with ionogel after healing. **b**, with aerogel and ionogel components stored half year in the glovebox. Source data are provided as a Source Data file.

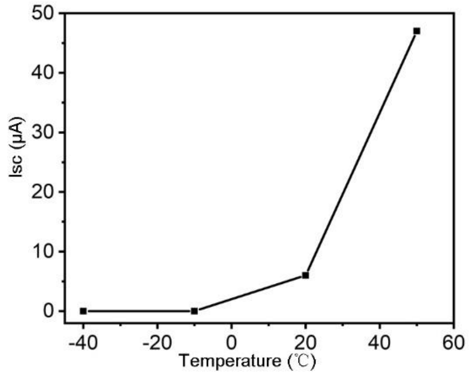

Supplementary Fig. 13 The output performance of the moisture-enabled GO aerogel-based power source without RTIL electrolyte under RH of 70% at different temperature. Source data are provided as a Source Data file.

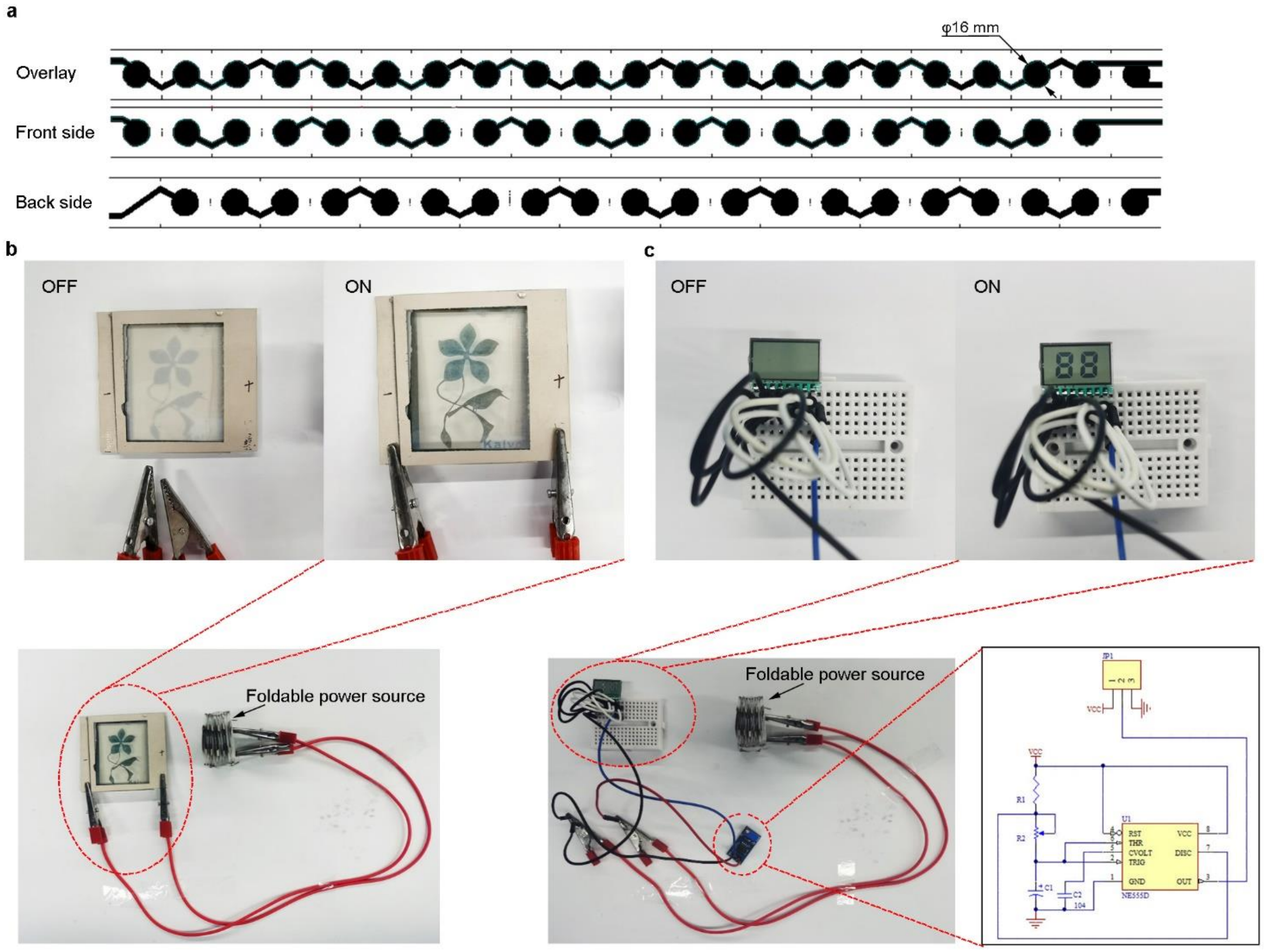

Supplementary Fig. 14 Demonstration of applications of GO aerogel osmotic power source with RTIL. a, The schematic of the foldable power source design with 20 cells in series connection on both sides of the substrate. **b,** The electrochromic device powered by a foldable power source. **c**, The liquid crystal display (LCD) screen powered by a foldable power source via the circuit based NE555 chip transfering DC to pulse current. The working voltage of the circuit is 5~12V.

References

1. Rollings, R. C., Kuan, A. T. & Golovchenko, J. A. Ion selectivity of graphene nanopores. *Nat. Commun.* **7**, 11408 (2016).
2. Kim, S. et al. Neuromorphic van der Waals crystals for substantial energy generation. *Nat. Commun.* **12**, 47 (2021).
3. Ji, J. et al. Osmotic Power Generation with Positively and Negatively Charged 2D Nanofluidic Membrane Pairs. *Adv. Funct. Mater.* **27**, (2017).
4. Sun, P. et al. Realizing synchronous energy harvesting and ion separation with graphene oxide membranes. *Sci. Rep.* **4**, 5528 (2014).
5. Wan, J. et al. Microwave Combustion for Rapidly Synthesizing Pore‐Size‐Controllable Porous Graphene. *Adv. Funct. Mater.* **28**, 180382 (2018).
6. Hong, S. et al. Two-Dimensional $Ti_3C_2T_x$ MXene Membranes as Nanofluidic Osmotic Power Generators. *ACS Nano* **13**, 8917-8925 (2019).
7. Zhang, Z. et al. Mechanically strong MXene/Kevlar nanofiber composite membranes as high-performance nanofluidic osmotic power generators. *Nat. Commun.* **10**, 2920 (2019).
8. Zhu, X. et al. Unique ion rectification in hypersaline environment: A high-performance and sustainable power generator system. *Sci.Adv.* **4**, eaau1665 (2018).
9. Zhang, Z. et al. Ultrathin and Ion-Selective Janus Membranes for High-Performance Osmotic Energy Conversion. *J. Am. Chem. Soc.* **139**, 8905-8914 (2017).
10. Guo, W. et al. Energy Harvesting with Single-Ion-Selective Nanopores: A Concentration-Gradient-Driven Nanofluidic Power Source. *Adv. Funct. Mater.* **20**, 1339-1344 (2010).
11. Kim, D.-K., Duan, C., Chen, Y.-F. & Majumdar, A. Power generation from concentration gradient by reverse electrodialysis in ion-selective nanochannels. *Microfluid Nanofluidics* **9**, 1215-1224 (2010).
12. Xin, W. et al. High-performance silk-based hybrid membranes employed for osmotic energy conversion. *Nat. Commun.* **10**, 3876 (2019).
13. Oh, J. M. et al. U1 snRNP regulates cancer cell migration and invasion in vitro. *Nat. Commun.* **11**, 1-8 (2020).

14. Gao, J. et al. High-performance ionic diode membrane for salinity gradient power generation. *J. Am. Chem. Soc.* **136**, 12265-12272 (2014).
