# Supplementary material for: Flexible iontronics based on 2D nanofluidic material
Source: Nat Commun. 2022 Aug 24;13:4965. doi: 10.1038/s41467-022-32699-x (PMC9402920; doi:10.1038/s41467-022-32699-x)
Supplement: Supplementary file 3 — Description of Additional Supplementary Files [file 41467_2022_32699_MOESM3_ESM.docx]

**Description of Additional Supplementary Files**

**File Name: Supplementary Movie 1**

**Description:** The energy generated from TENG could power a 1.5 V LED.

**File Name: Supplementary Movie 2**

**Description:** The Origami inspired foldable osmotic power source from pure ion gradient can power the electrochromic device.
